# Spontaneous formation of graphene-like stripes on high-index diamond C(331) surface

**DOI:** 10.1186/1556-276X-7-460

**Published:** 2012-08-16

**Authors:** Maojie Xu, Yaozhong Zhang, Jing Zhang, Jiyun Lu, Bingjian Qian, Dejiong Lu, Yafei Zhang, Liang Wang, Xiaoshuang Chen, Hidemi Shigekawa

**Affiliations:** 1 Key Laboratory for Thin Film and Microfabrication of the Ministry of Education, Research Institute of Micro/Nano Science and Technology, Shanghai Jiao Tong University, Shanghai, 200240, People’s Republic of China; 2 National Laboratory for Infrared Physics, Shanghai Institute of Technical Physics, Chinese Academy of Sciences, Shanghai, 200083, People's Republic of China; 3 Institute of Applied Physics, University of Tsukuba, Tsukuba, 305-8573, Japan

**Keywords:** Surface reconstruction, Density functional theory, Graphene, Diamond, 68.35.bg, 68.47.Fg, 68.35.Md

## Abstract

We employ first-principles density functional theory calculations to study the surface reconstruction, energetic stability, and electronic structure of diamond C(331) surface. Spontaneous formation of graphene-like stripes on the reconstructed surface is found to occur as the surface terrace C atoms transform from *sp*^3^ to *sp*^2^ hybridization upon structural relaxation. The comparison of the calculated absolute surface energies of C(331), C(111), and C(110) surfaces demonstrates the energetic stability of the graphitic-like C(331) surface. Local density of electronic states analysis reveals the occurrence of localized electronic states near the Fermi level, which may have a significant impact on the surface conductivity.

## Background

Diamond holds a variety of extraordinary physical and chemical properties, facilitating its possible applications in novel functional devices [1-7]. As a semiconductor with a wide bandgap of 5.47 eV, it is a promising candidate for short-wavelength optoelectronic devices such as ultraviolet light-emitting diodes. The extreme mechanical hardness of diamond endows it with potential applications in nanomechanical devices. When doped with boron, it was found to display superconductivity around liquid helium temperature. To utilize the qualities of diamond, it is imperative to grow high-quality materials. Chemical vapor deposition is an efficient and versatile technique for the growth of diamond. A large body of experiments and theories are dedicated to understanding the growth process [8]. Graphitic-like surface reconstructions on stepped C(111) surfaces are predicated by first-principles calculations [9]. Surface graphitization of diamond nanoparticles is investigated from an experimental viewpoint [10]. A unique character of diamond growth is the existence of *sp*^2^-hybridized bonds in the graphitic-like layer of diamond surfaces, in contrast to other group IV element semiconductors (Si and Ge), which do not exhibit energetically favorable *sp*^2^ bonding configurations. This may account for different surface reconstructions on Si and diamond surfaces [11]. Besides low-index surfaces, high-index Si surfaces are extensively investigated to unveil their atomic and electronic structures [12,13], whereas less attention has been paid to the study of high-index diamond surfaces. The graphite-like *sp*^2^ bonding is expected to give rise to the significant difference between high-index diamond and Si surfaces.

Graphene, a two-dimensional atomic crystal with graphite-like *sp*^2^ bonding, has attracted considerable interests due to its novel physical and chemical properties and its potential applications in nanoelectronics and optoelectronics [14]. Large-scale graphenes are grown on metal substrates [15]. Here, we explore the formation of graphene-like stripes on a reconstructed high-index diamond C(331) surface using first-principles density functional theory (DFT) calculations. During the structural relaxation of the bulk-terminated surface, the terrace C atoms in the first layer delaminate from the second layer, leading to local *sp*^3^ to *sp*^2^ rehybridization and the formation of graphene-like stripes on the surface. The driving force for the graphitic-like reconstruction is the presence of high-density dangling bonds on the surface, which gives rise to the rebonding of top-layer atoms. The comparison of the calculated absolute surface energies of C(331), C(111), and C(110) demonstrates the relative stability of the C(331) surface with the graphitic-like reconstruction. Local density of electronic states (LDOS) analysis reveals the occurrence of localized electronic states near the Fermi level (FL), which may play an essential role in determining the surface conductivity [16,17].

## Methods

The calculations are conducted in the framework of the DFT method by DMol^3^ codes [18]. We use the Perdew-Burke-Ernzerhof generalized gradient approximation [19]. A double numeric basis set including *d*-polarization function, all electron treatment, and an 8 × 2 × 1 Monkhorst-Pack *k-*point mesh for the Brillouin zone sampling [20] are employed to carry out geometry optimization and electronic band structure calculations. Spin-unpolarized self-consistent field calculations are performed with a convergence criterion of 2.0 × 10^−5^ hartree (1 hartree = 27.2114 eV) for total energies. The maximum force tolerance is 0.004 hartree Å^−1^, and the maximum displacement tolerance is 0.005 Å.

**Figure 1:**
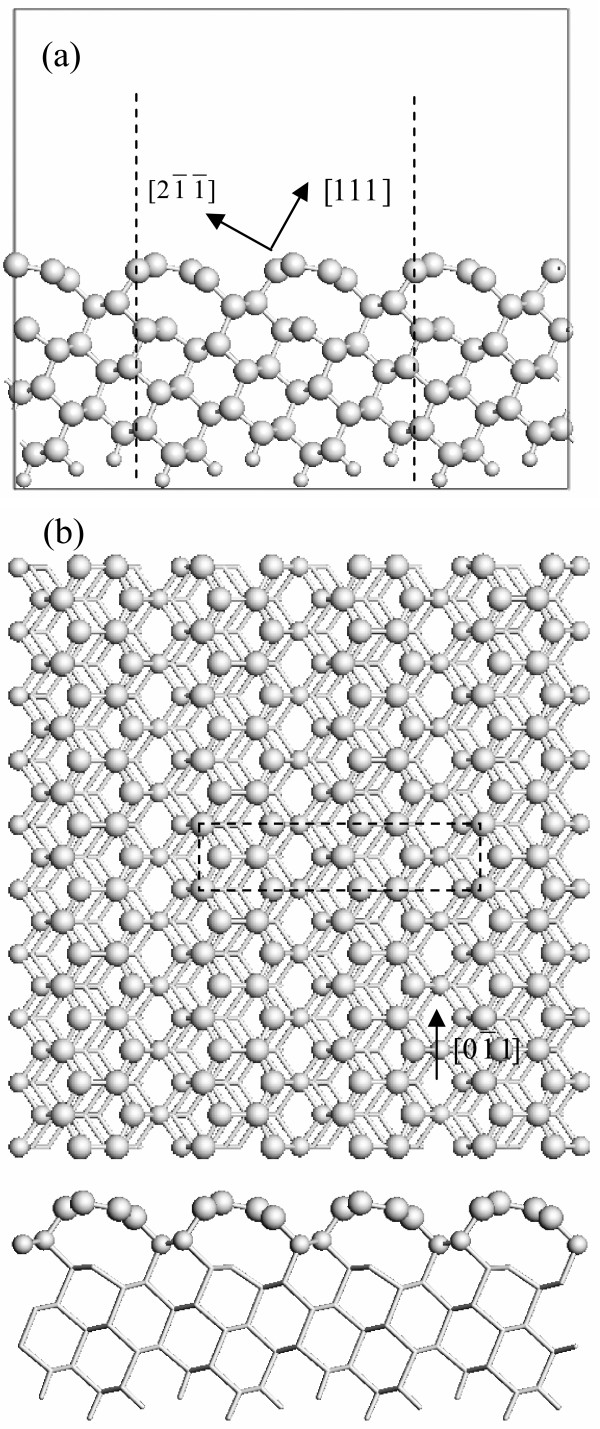
**Calculated atomic structure of diamond C(331) surface with graphene-like stripes.** (**a**) The dashed lines indicate the supercell viewed from the $\left[0\bar{1}1\right]$ direction. The large circles denote the C atoms, and the small circles denote the H atoms. (**b**) The dashed box indicates the supercell viewed from the [331] direction, and the bottom is viewed from the $\left[0\bar{1}1\right]$ direction. The large circles denote the C atoms of the graphitic layer, and the smaller circles indicate the *sp*^3^-bonded C atoms in the outmost surface. The other C and H atoms are represented by the smallest circles.

**Figure 2:**
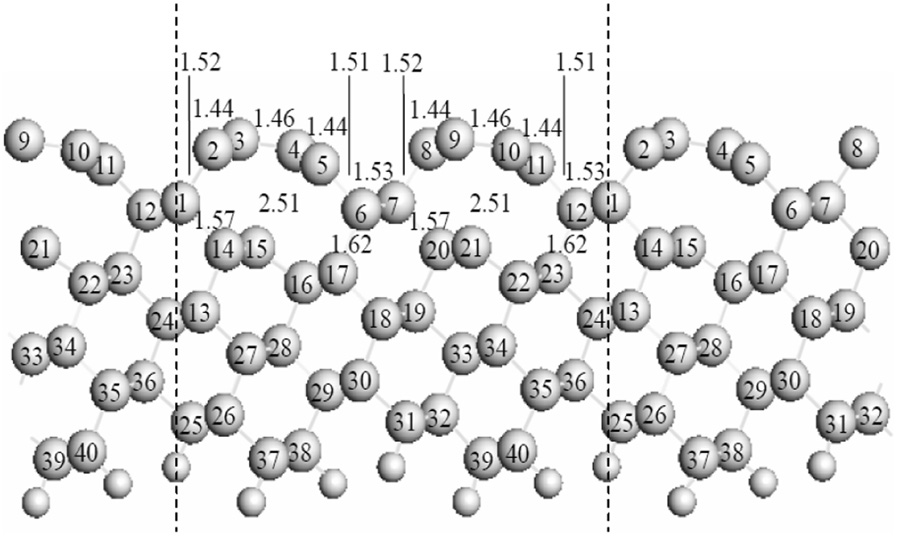
**Calculated atomic structure of H-passivated C(331) (1 × 1) surfaces.** The dashed lines indicate the supercell viewed from the $\left[0\bar{1}1\right]$ direction. The large circles denote the C atoms, and the small circles indicate the H atoms.

The periodically repeated slabs separated by approximately 10 Å of vacuum are used to represent the surface structures. Each slab of C(331) surface is composed of 11 atomic layers with 40 C atoms and 6 H atoms per unit cell. The H atoms are used to passivate the surface C atoms at the bottom of the slabs to make the calculation more efficient. The dashed lines in Figure 1a and the dashed box in Figure 1b indicate the supercell used for the calculation. Each slab of H-passivated C(331) surface is composed of 12 atomic layers with 40 C atoms and 12 H atoms per unit cell. The dashed lines in Figure 2 indicate the supercell used for the calculations.

## Results and discussion

**Figure 3:**
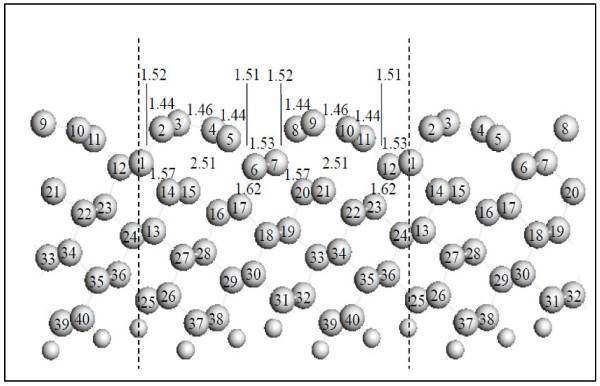
**Representative structural parameters of C(331) surface with the graphene-like stripes viewed from the**$\left[0\bar{1}1\right]$**direction.** Interatomic distances are given in Ångström. The large circles denote the C atoms, and the small circles denote the H atoms.

Figure 1 shows the atomic structure of the graphene-like stripes formed on the reconstructed diamond C(331) surface calculated after the structural relaxation of the bulk-terminated surface. We allow this surface to relax using a steepest descent algorithm. The top-layer C atoms exhibit the *sp*^2^ bonding configuration in the graphene-like structure, as shown in Figure 1b. Upon structural relaxation, the terrace C atoms (see 4 and 10 C atoms in Figure 3) delaminate from the subsurface diamond and form the graphene-like stripes along the $\left[0\bar{1}1\right]$ direction. The energetically favorable hexagonal rings are found to emerge in the graphitic layer on the reconstructed surface. The driving force for the graphitic-like reconstruction on the surface is the presence of high-density dangling bonds which have unpaired electrons. This situation is similar to the reconstruction of the C(111) surface, where the top-layer C atoms are rearranged to make the dangling bonds become the nearest neighbors and form the π bonding [21]. For the C(331) surface, the delamination of the terrace C atoms can lead to the formation of graphite-like *sp*^2^ bonds, thereby reducing the energetically unfavorable dangling bonds.

The representative C-C bond lengths for the graphitic-like reconstructed C(331) surface are shown in Figure 3. The distance between the delaminated C atom and the subsurface C atom increases to approximately 2.51 Å, much larger than the bond length of diamond (1.54 Å). The bond lengths for the C atoms in the graphitic structure decrease to 1.44 and 1.46 Å. These values are quite close to the bond length of graphite (1.42 Å), whereas much smaller than that of diamond. The C atoms with the unsatu-rated dangling bonds at the subsurface positions remain *sp*^3^-hybridized in character, although they have stretched by almost 34%. The C-C bonds are stretched to 1.62 and 1.57 Å for the outmost C atoms attached to the second-layer C atoms. The severe subsurface rebonding increases the elastic strain, which is energetically unfavorable. The competition between the favorable *sp*^2^ bonding in the graphitic layer and the unfavorable strain energy leads to the graphitic-like reconstruction of the C(331) surface.

The energetic stability of the C(331) surface is studied by comparing its absolute surface energy (ASE) with those of low-index diamond C(111) and C(110) surfaces [21-23]. In the centrosymmetric slab used for computing the ASE, the top and bottom surfaces are physically equivalent. After full structural relaxation, the same *n* × *m* surface reconstruction is observed to occur on both sides of the slab. Therefore, it allows calculating directly the ASE. For the slab with *N* atoms at the atomic configuration $\left\{R_{i}\right\}$, the surface energy per 1 × 1 surface cell, $E_{\text{surf}}^{n\times m}$, can be calculated from the total energy $E_{\text{tot}}\left(N,\left\{R_{i}\right\}\right)$ of the slab subtracted by *N* times the bulk diamond energy μ per atom. The surface energy is expressed as

(1) $$E_{\text{surf}}^{n\times m}=\frac{1}{2nm}\left\{E_{\text{tot}}\left(N,\left\{R_{i}\right\}\right)-N\mu \right\}\text{.}$$

Since two equivalent surfaces are involved in the calculations for a slab, a prefactor, $\frac{1}{2}$, is added in Equation 1. For the *n* × *m* surface reconstruction, the *nm* gives the number of the 1 × 1 surface cell. The surface energy per unit area is as follows:

(2) $$\gamma^{n\times m}=\frac{E_{\text{surf}}^{n\times m}}{A}\text{,}$$

where *A* is the area of a 1 × 1 surface cell for a given surface orientation *n*. For the H-covered C(331) surface, the surface energy per 1 × 1 surface cell is given by

(3) $$E_{\text{surf}}^{\text{H}}=\frac{1}{2}\left\{E_{\text{tot}}\left(N,N_{\text{H}},\left\{R_{i}\right\}\right)-N\mu -N_{H}\mu_{H}\right\},$$

where $E_{\text{tot}}\left(N,N_{\text{H}},\left\{R_{i}\right\}\right)$ is the total energy of the slab, N_H_ is the number of H atoms, and μ_H_ is the chemical potential of the H atom in the reservoir that is defined in [21]. Table 1 collected the surface energies $E_{\text{surf}}^{n\times m}$, $\gamma^{n\times m}$, and $E_{\text{surf}}^{H}$ for various orientations and reconstructions. The computed energies for low-index C(111) and C(110) surfaces agree well with the previous investigation [21]. The graphitic-like reconstructed C(331) surface is found to have lower $\gamma^{n\times m}$ than low-index C(111) and C(110) surfaces, indicating that the C(331) surface is one of the stable crystalline diamond surfaces.

**Table 1:** **Absolute surface energies**$E_{\text{surf}}^{n\times m}$**and**$\gamma^{n\times m}$**for various orientations and reconstructions**

**Orientation**	**Reconstruction**	***E***_**surf **_**(eV/1 × 1 cell)**	***γ *(J/m**^**2**^**)**
(111)	2 × 1	0.993	2.91
		(1.369)	(4.06)
	H-covered	−1.903	−5.57
		(−2.760)	(−8.19)
(110)	1 × 1 relaxed	1.824	3.27
		(3.264)	(5.93)
	H-covered	−4.971	−8.91
		(−5.496)	(−9.99)
(331)	1 × 1 graphitic	2.040	2.31
	H-covered	−5.808	−6.58

The values in parentheses from [21] are listed for comparison.

The H adsorption on the graphitic-like reconstructed C(331) surface is found to give rise to the reversion of *sp*^2^ hybridization back to *sp*^3^ hybridization. Figure 2 shows the calculated atomic structure of the H-covered C(331) (1 × 1) surface. The top-layer C atoms display *sp*^3^ bonding configuration. Thus, the H atoms can give rise to the dereconstruction of the graphitic-like C(331) surface.

**Figure 4:**
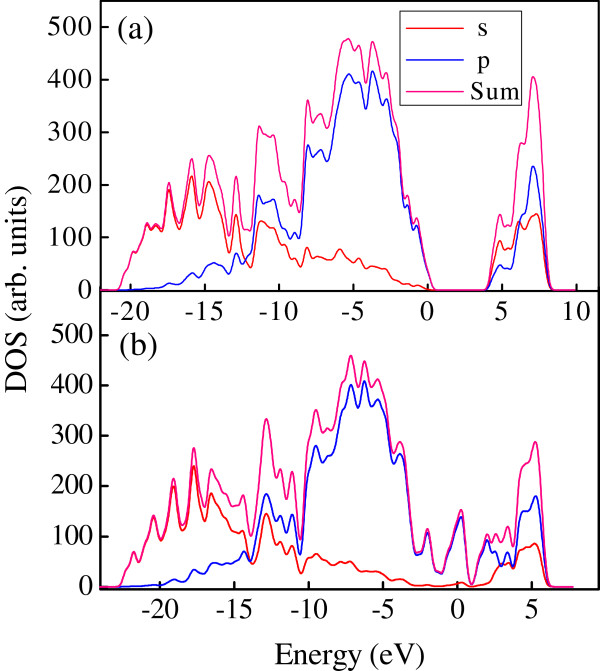
**LDOS and PDOS of (a) H-passivated and (b) graphitic-like reconstructed C(331) surfaces.** The zero energy corresponds to the FL. The peak near the FL in the LDOS curve of (**b**) is associated with the localized electronic states at the surface and subsurface regions, which may have a significant impact on the surface conductivity.

Figure 4a shows the LDOS of the H-passivated diamond (331) surface. The zero energy corresponds to the FL which is at the position of the top valence band. An energy bandgap of 4.2 eV is obtained from the calculated electronic band structure. Figure 4b shows the LDOS of the reconstructed C(331) surface with the graphene-like stripes. The zero energy corresponds to the FL, which lifts up to a position in the bulk bandgap. The peak near the FL in the LDOS curve is attributed to the localized electronic states at the graphitic surface and subsurface regions, which may give rise to the semimetallic or metallic conduction along the surface. Further partial electronic density of states (PDOS) analysis reveals that the localized electronic states near the FL is predominant with the *p* character for the graphitic-like reconstructed C(331) surface.

## Conclusions

We carry out first-principles DFT calculations to study the spontaneous formation of graphene-like stripes on the reconstructed diamond C(331) surface. The *sp*^2^-hybridized bonding in the graphitic layer on the surface plays a central role in reducing the energetically unfavorable dangling bonds on the bulk-terminated surface, thereby lowering the surface free energy. A sharp peak is found to occur near the FL in the LDOS curve, which arises from the localized electronic states at the surface and subsurface regions. These states may have a significant impact on the surface conductivity. The graphene-like stripes directly formed on a semiconductor surface may be used for nanoelectronic and optoelectronic devices.

## Abbreviations

ASE: absolute surface energy; DFT: density functional theory; FL: Fermi level; LDOS: local density of electronic states; PDOS: partial electronic density of states.

## Competing interests

The authors declare that they have no competing interests.

## Authors’ contributions

MJX did the calculations and wrote the manuscript. YFZ conceived and suggested the calculations. YZZ, JZ, BJQ, JYL, DJL, LW, XSC, and HS discussed about the calculations and revised the final manuscript. All authors read and approved the final manuscript.

## Authors’ information

Dr. MJX obtained his Ph.D. from University of Tsukuba, Japan, and is currently working with Prof. YFZ as postdoctoral research fellow in Shanghai Jiao Tong University, China. Mr. YZZ, Ms. JZ, Mr. BJQ, Mr. JYL, and Mr. DJL are currently postgraduate students in Shanghai Jiao Tong University. Dr. YFZ obtained his Ph.D. from Lanzhou University, China, and is currently working as a professor in Shanghai Jiao Tong University. Dr. LW obtained his Ph.D. from Shanghai Institute of Technical Physics, Chinese Academy of Sciences, China, and is working with Prof. YFZ as postdoctoral research fellow. Dr. XSC obtained his Ph.D. from Nanjing University, China, and is currently working as a professor in Shanghai Institute of Technical Physics, Chinese Academy of Sciences, China. Dr. HS obtained his Ph.D. from Tokyo University, Japan, and is currently working as a professor in University of Tsukuba, Japan.
